# How can we promote and facilitate effective study skills in medical students?

**DOI:** 10.15694/mep.2017.000036

**Published:** 2017-02-22

**Authors:** Sebastian Charles Keith Shaw

**Affiliations:** 1 Brighton and Sussex Medical School

**Keywords:** Study Skills, Medical Students, Undergraduate, Learning to Learn

## Abstract

This article was migrated. The article was marked as recommended.

In my early years of medical school I always felt that I never learnt how to learn. When studying an MSc in Medical Education, it became apparent to me that this feeling may be commonplace amongst other medical students.

Study skills can be defined as “the effective use of appropriate techniques for completing a learning task..” These are of vital importance in any educational setting - allowing learners to tackle and absorb information appropriately and efficiently. Although study skills are recognised as an essential ability, they may be overlooked by many medical curricula. This paper therefore aims to provide insight into the self-directed learning process and the development of appropriate study skills.

The self-directed learning process is discussed - using an adapted form of the Self-directed Learning Cycle of Butler and Winne. This involves: the domains of learning; identifying learning deficits; setting learning goals; identifying efficient learning strategies; monitoring progress and strategy modification; and reaching goals. I also discuss the importance of learning style recognition, maintaining the desire to learn and possible methods for the assessment of study skills in medical students.

Effective study skills are a vital tool in the medical student’s arsenal against the vast quantity of information presented to them. These allow the learner to appropriately filter and assimilate information. When both understood and mastered, an individual can adapt these to the fit needs of various knowledge requirements.

Through the understanding of memory and the core principles of learning, educators can hope to promote and facilitate the development of such study skills. In doing this, we can help our students to become self-sufficient, effective members of the medical community.

## Introduction

As a medical student I have always felt that I never learnt how to learn. This has been particularly challenging for me due to my own struggles with dyslexia. Throughout my teaching experiences, and studying an intercalated Masters Degree in Medical Education, it has become apparent to me that this feeling is commonplace. I would therefore like to explore how we may aid our learners in developing the necessary skills to undertake effective learning.

Study skills can be defined as “the effective use of appropriate techniques for completing a learning task.. We distinguish between ‘study skills’ and ‘study techniques.’ A study technique is a particular procedure used to perform a learning task. A study skill is the ability to use that technique appropriately and efficiently”(1). These are of vital importance in any educational setting; allowing learners to tackle and absorb information appropriately and efficiently.

Although study skills are recognised as an essential ability, they may be overlooked by many medical curricula (2). For this reason, I would like to consider:

“How can we promote and facilitate effective study skills in medical students?”

Through better understanding of the ways in which people learn and revise, educators might adapt the ways in which we facilitate this to maximise our efforts.

## Core Principles of Learning

Learning is not a single or linear process. It is in fact a series of events and may be divided into two broad categories: active learning and passive learning (3). These categories may be further subdivided, however this is not necessary for a basic understanding with regards to education. Active and Passive learning are outlined in Table 1.

**Table 1.** Overview of Active and Passive Learning

Active Learning	Passive Learning
Generating your own learning strategies.	Waiting for specific instruction.
Questioning in order to gain understanding.	Blindly accepting facts and transcribing notes.
Using feedback to adapt and improve learning.	Ignoring feedback.
Using multiple sources to learn and discover aspects that are of particular interest to you.	Doing the bare minimum to pass summative assessments.

Table 1: Active and Passive Learning. Adapted from Moore at al (3).

By necessity, passive learning is commonplace within medical education - particularly in the early years of study (2). Active learning may be promoted more heavily as learners progress through the years of study (2). Regardless of the learning method, there are several core domains into which learning can be classified. These are outlined in Table 2.

**Table 2.** Core Domains of Learning

Domain	Description
Cognitive	Learning about things (knowledge)
Psychomotor	Learning to do things (skills)
Affective	Developing personally (attitudes)
Psychosocial	Learning to work in a team (social skills)

Table 2: Core Domains of Learning. Created using information from Royal College of Psychiatrists (4)

It could be argued that strength in all four domains is a necessity for doctors and should therefore be developed during a learner’s time at medical school. For this reason, it is essential to include either training in these domains, or tasks promoting their development within a medical curriculum - be it via active or passive methods.

## The Basic Concept of Memory

Learning and memory are heavily entwined as concepts. After all, how can an individual learn without the ability to remember? Memory can be thought of as “the retention of learned information” (5). Once again this can be split into two broad subtypes: declarative and non-declarative memory (5, 6).

Declarative memory is what is perhaps traditionally thought of as memory (6). This refers to the storage of facts, figures and events (5). This is therefore vitally important for the study of Medicine. Non-declarative memory however relates to the storage of procedural and emotional content (5). For example, this may include the ability to ride a bicycle or a learned fear of snakes. A core difference between the two forms is the way in which they are recalled. Declarative memories can be consciously recalled whereas non-declarative cannot (5, 6).

For the purpose of this paper I am primarily focusing on learning in relation to declarative memory, as this may hold the most bearing to medical education. It could however be argued that, in some form, both types play important and overlapping roles.

## Self-Directed Learning

Self-directed learning (SDL) personifies active learning in its truest form (8). Many people may think of study skills and SDL as synonymous terms, however this is not the case. SDL refers to *“a process by which learners manage their own learning process from beginning to end”* (8). Study skills are therefore a core element of SDL, but should be thought of as key transferable skills which are not restricted to this subset of learning.

As doctors of the future, medical students will be expected to develop the ability to locate and assimilate new Information in a self-sufficient manner (2). It is therefore imperative that the required skills be nurtured throughout their time at medical school.

**Figure 1.**
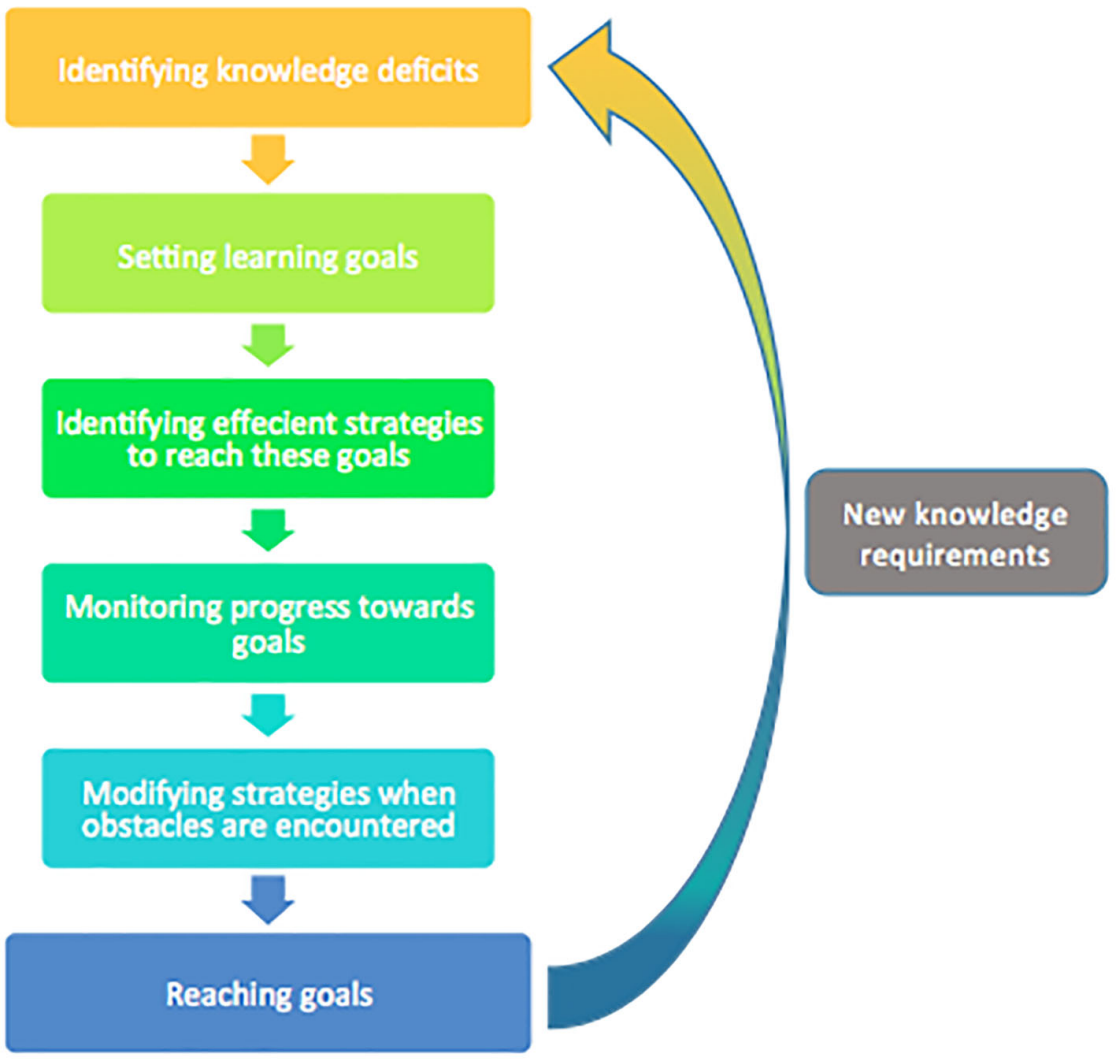
Self-Directed Learning Cycle. Created using adapted information from Butler and Winne (10).

SDL requires the learner to move through a series of stages which form a cyclical process as an increasing amount of information is needed (2, 3, 9). An adapted version of this process is shown in Figure 1. The steps within this model have been heavily adapted from the feedback-based model of Butler & Winne in order to be applied to SDL.

In order to promote this method of SDL, it could be argued that we should provide learners with training in study skills related to SDL - especially in the early years of study. Once this is complete, we may then facilitate revalidation, refinement and individualisation of these skills by setting appropriate tasks for our learners. Throughout the forthcoming sections, it is my intention to detail our role within each of the steps of the above cycle ( Figure 1).

## Identifying Learning Deficits

Once a new knowledge requirement has been encountered, a key part of the learning process is identification of your knowledge deficits (2). There are several ways in which this could be achieved, and we can facilitate these as medical educators.

It is our duty as educators to provide clear and specific Learning Objectives (LOs) for our teaching (11). In making our learners aware of the LOs for each session, we might hope to present them with the ‘knowledge requirement’ stimulus. They may then use our LOs to identify deficits in the required knowledge and begin planning the learning process.

Another valuable method in which we may assist our learners is through the use of formative assessment (2, 12). In setting these, we may provide an accessible opportunity for our learners to self-assess their understanding of selected topics (12). Thereby paving the way for them to commence both learning and revision.

*“Formative assessment is designed to aid learning by generating feedback information that benefits students during the learning process and leads to enhanced learning outcomes”* (12).

Peer discussion has also been suggested as a way of initiating the process (2). This can allow learners to gage their level of understanding against those at the same level (2). It may also promote competition and therefore provide further incentive to learn.

One potential strategy to combine the above methods may be to run regular Problem-Based Learning (PBL) sessions (13). In facilitating these, we could provide LOs, introduce peer discussion and incorporate formative assessment. Such sessions could also potentially help learners in the Goal Setting stage. PBL sessions have also been shown to improve the application of knowledge within clinical environments (13).

## Why Help Learners Develop Study Skills?

Miller showed the benefits of addressing poor study skills before these inappropriate study skills are developed (7). In doing this, we can hope to prevent learners from becoming heavily reliant on such study skills and decompensating at a later stage.

## Setting Learning Goals

In this stage of the cycle, students must choose a realistic expectation of learning outcomes in relation to the quantity and difficulty of the information (2). We may facilitate the formation of these goals by clearly showing our expectations within LOs and lesson aims.

## Identifying Efficient Learning Strategies

There are numerous strategies to promote and maximise the efficiency of learning. Many of these tackle the way in which we present information to ourselves. There are however some strategies which involve changing our attitudes towards both learning and the content we wish to assimilate (9). To be truly efficient, the strategies adopted should be task-dependant and adaptable to the needs of the exercise (2, 3, 9).

‘Chunking’ refers to breaking down information into small, manageable parts (14). Studies have shown this to be beneficial to the learning process (15). Therein lies its potential for our manipulation. When teaching, we may attempt to chunk the information we impart into small and logical segments. In doing this, we might hope to further facilitate the learning process.

It has also been shown that applying emotional significance to a topic increases an individual’s ability to store and recall the information (16). It could be argued that this emotional significance allows learners to bridge declarative and non-declarative memory. Therefore using non-declarative processes to aid storage whilst using the declarative system to allow conscious recall for examinations and beyond. This efficiency can be further improved by applying personal meaning to new information (17).

### Concept Mapping

A fast-emerging strategy in Medical Education is the idea of concept mapping (18). These are more than simple mind maps. A concept map “is a device for representing a set of concept meanings embedded in a framework of propositions.” (19)

These could allow learners to gain an understanding of how individual topics fit into the broader spectrum of information (18). This is because they are designed to show direct associations between new knowledge and that which is already known (19). Teaching this strategy to learners could promote long-term retention of the information learned (18).

These might be of particular use when medical students enter the clinical phase of their training. As this stage of medical curricula may require students to gain a wide understanding of medical subjects and their interactivity.

### Critical Appraisal Skills

A key element of study skills, especially as part of SDL, is the ability to filter the vast quantity of information available (2). For this reason, the ability to critically appraise information sources is a vital part of medical education (20).

There are many ways in which we could teach this to learners. Once such strategy is through use of critical appraisal checklists. A forerunner of this approach is Oxford’s Critical Appraisal Skills Programme (CASP) (21). By signposting learners to such a resource, we may allow them to develop appropriate strategies of their own.

## Monitoring Progress and Strategy Modification

Once learners have set goals and adopted a strategy to reach them, it is important that they continually assess the effectiveness of their strategy (22). In doing this, they could both maximise their likelihood of reaching these goals and the efficiency with which they do so (3, 9). This is important, as learners should be able to manipulate revision strategies to meet their individual needs, not the other way around (9). Although it may seem like we have no role to play within is stage, formative assessment could be used to gage strategy effectiveness (12). This might begin to tackle the various limitations and weaknesses associated with the self-assessment process (22).

## Reaching Goals

Attainment of learning goals is the desired outcome for any learning or revision activity. This may however not be possible. Therefore goal modification may be required to create more realistic expectations of learning capabilities within given time constraints (2). Through scheduled use of summative assessment methods, we can provide the necessary time constraints for learners to undergo the learning process and reach their goals (23). Such assessments would also validate whether or not these goals had been reached - both to the learners and to us as educators (23).

## Desire to Learn

Although the above sections detail our role in facilitating the learning process, the mention of promotion is relatively superficial. In order for learners to develop appropriate study skills, they must first have a desire to learn (17). Generating this desire could be considered core to the promotion of effective study skills. Bandaranayake highlights the importance of finding an incentive to learn information (2). This once again revalidates the importance of clear and logical LOs.

It has however been demonstrated that motivation to learn may not directly affect our ability to learn (24). It undoubtedly promotes the learning process nonetheless (17). This may be through indirect measures such as increased time spent on the learning activity (17).

Elective learning refers to the concept of allowing learners to choose the topics they study (2). In allowing students to choose what they learn, we might maximise the quantity and efficiency of their learning (2). Although this may be constrained by core knowledge requirements, there may still be opportunities to encompass this within medical curricula. For example, a medical school might offer a selection of study choices which complement a core module (25).

## Recognition of Learning Style

Questionnaires such as Kolb’s Learning Style Inventory (LSI) can be used to identify people’s learning styles (26). It is however important for students not to become heavily reliant on the results of such a test (9). In doing this there would be a risk of learners committing themselves fully to a reduced spectrum of leaning strategies (9). It is therefore more important that learners experiment with various strategies to discover individualised approaches that fit their needs - using these results to attempt previously unconsidered techniques.

## Assessment of Study Skills in Learners

Although we may pour significant effort into the promotion and facilitation of appropriate study skills, it is important to assess if our efforts are well-placed (23). Within this section I aim to highlight and critique several methods with which this goal could be achieved.

One potential method is through use of formative assessment (12). This could allow us to gage learners’ readiness for approaching summative assessments (12, 23). Unfortunately, due to its formative nature, this may not provide the necessary incentive to have commenced revision strategies, let alone to have completed them.

Another option is through use of summative assessments. These could provide the incentive for learners to try due to the recognised importance of the assessment (23). A series of such assessments may also allow us to locate learners who are consistently struggling with the academic workload (23). Thereby providing us with the opportunity to intervene. Unfortunately, this method may disadvantage such students as, in some ways, intervening after summative assessments could be considered too late.

In a keystone of educational research, Jones & Slate created the Study Habits Inventory (SHI) questionnaire (27). This provides a method for directly assessing the study skills of learners without the confounder of Medical Content being assessed (27). This could however prove a costly and time-consuming method to implement alongside the main curriculum.

Although none of the above methods are perfect, a combination could potentially be used to gage the study skills of medical students throughout the training process. In doing this, we could hope to promote the steady development of study skills whilst using our summative assessments to catch any struggling learners who may have otherwise slipped through unnoticed.

## Further Considerations

### The Importance of Feedback

Feedback has been shown to be vital in the learning process (12). This allows learners to adapt their study skills for future learning tasks and assessments (12). In taking the time to provide detailed feedback on performance, we might further drive the development and refinement of appropriate study skills.

### Near-Peer Teaching

A final consideration might be the very teachers we use to educate our learners. Studies have shown Near-Peer Teachers (NPTs) to be a highly effective educational tool (12). It has been suggested that this is due to better teacher-learner communication (12). NPTs may also be able to impart knowledge and skills at a more appropriate level (28). For these reasons, NPTs may be worth serious consideration in any study skills educational intervention.

## Proposed Solution

I propose one of numerous potential solutions. This solution may relate most effectively to medical schools adopting a spiral curriculum due to suggestion of repetition.

Medical students could have their study skills assessed on arrival at university with the SHI or an equivalent - they may also benefit from assessment with Kolb’s LSI. The results should then be provided to the students so that they might gain a baseline understanding of their abilities. They may then be taught a selection of study skills within a short programme of sessions - with inclusion of NPTs. These should be located as early in the curriculum as possible - preferably within the first ‘term’ of study. Within these sessions, the importance and personal relevance of topics covered should be stressed. The medical students could be taught key study skills, such as:

• Academic writing (3).
• Searching the literature and critical appraisal (see above).
• Principles of SDL - including explanation of a cycle applied to their current learning (see above).
• Study scheduling / revision diaries (3, 9).
• Concept mapping (see above).

The above sessions could then be offered, optionally, in later years of study to refresh and revalidate learners’ study skills. Furthermore, regular formative assessments may be undertaken to drive and facilitate the learning process (12).

Summative assessments requiring a variety of study skills, such as examinations and essays, should be utilised to force further development of these abilities. In making such assessments summative, we can hope to provide the necessary incentive for both the content and skills gained to be committed to memory (23).

Furthermore, PBL sessions could be conducted on one or two occasions each week throughout the academic years of study. These would allow the learners to practise a variety of study skills in a guided manner (13). Thereby preventing development of a heavy reliance on ineffective study habits.

## Conclusions

Effective study skills are a vital tool in the medical student’s arsenal against the vast quantity of information presented to them. These allow the learner to appropriately filter and assimilate information. When both understood and mastered, an individual can adapt these to the fit needs of various knowledge requirements.

Through the understanding of memory and the core principles of learning, educators can hope to promote and facilitate the development of such study skills. In doing this, we can help our students to become self-sufficient, effective members of the medical community.

## Take Home Messages

• Study skills are an important aspect of medical education.
• They may be overlooked by many medical curricula.
• Understanding the basic theories of memory and learning may aid us in the promotion of effective study skills.
• I propose that an early study skills programme may enhance the ability of medical students to develop them - repeated at later stages in their education, to promote retention.

## Notes On Contributors

**Sebastian Shaw**, MSc, MAcadMEd, AFHEA, is a final year medical student. His main interests in education are dyslexia and the social psychology of the student/trainee experience. During 2014/15 he undertook an MSc in Medical Education, which also sparked his interest in the development of study skills.
