# Supplementary material for: How can we promote and facilitate effective study skills in medical students?
Source: MedEdPublish (2016). 2017 Feb 22;6:36. [Version 1] doi: 10.15694/mep.2017.000036 (PMC10885237; doi:10.15694/mep.2017.000036)
Supplement: Peer review history

# Reviewer report: Reviewer response for version 1

**DOI:** 10.21956/mep.19441.r28613

**Published:** 2017-02-22

**Authors:** Trevor Gibbs

**Affiliations:** 1 AMEE

This review has been migrated. The reviewer awarded 4 stars out of 5 An interesting paper that I enjoyed reading. I don't feel that there was too much in the paper that could not be found in standard medical education textbooks, but I felt that it was a god summary of some well accepted approaches plus some personal thoughts. There were a few points that I felt a little uncomfortable with:i) On the question of PBL and Learning Objectives (Los), I have seen so many schools that use PBL and give the students the Los before the session begins- this I think leads to cutting corners, superficial learning and moves away from one of the basic principles which is about finding the gap between what the students know presently and what they don't know; found through discussion and exploration.ii) There is a great problem I feel with Self Directed Learning in that the students don't always know what they don't know, and as the author points out, have little ability to find out. Hence I would prefer the use of Directed Self Learning. The teacher can open the door to learning but acts as a guide to take the student through the door.iii) I think that there is now enough literature out there to suggest that Learning Styles are as black and white as we previously thought; more that we adopt our learning style to suit what we are learning. We may have preferences but we don't / can't always use that style.As the author points out, learning how to learn is often missing from the curriculum; finding a place for that activity within the curriculum is not that easy

Reviewer Expertise:

NA

No decision status is available

# Reviewer report: Reviewer response for version 1

**DOI:** 10.21956/mep.19441.r28614

**Published:** 2017-02-22

**Authors:** Richard Hays

**Affiliations:** 1 James Cook University

This review has been migrated. The reviewer awarded 3 stars out of 5 This is a useful and digestible summary of an interesting topic. Learning style are important, although are more of a discussion point than a rigid categorisation, Educators should be wary of forcing learners through a particular curriculum design out of their comfort zones. While I am a fan of helping students understand education, formal inclusion in already crowded curricula might not work well unless the students are 'ready' for the messages. There are many ways to learn and many learning resources available, and most learners work this out. Perhaps it is the 'working this out' part that is the important goal. I would agree that medical programs may be doing a great service if they could help their students gain self-awareness of their personal preferred learning styles. Most published studies suggest that medical students like small group, interactive learning (but some dislike this), and there is a move towards attending remotely and asynchronously via information technology. Further, many medical schools have 'underground' learning resources compiled by students over the years, providing the 'core' content that appears in examinations. Regardless of what teachers do, learners find ways to satisfy the learning outcomes that we measure. How can 'learning power' best be enhanced by educators and institutions?

Reviewer Expertise:

NA

No decision status is available
